# ARP-1 Regulates the Transcriptional Activity of the Aromatase Gene in the Mouse Brain

**DOI:** 10.3389/fendo.2020.00306

**Published:** 2020-06-03

**Authors:** Shin-ichiro Honda, Nobuhiro Harada

**Affiliations:** ^1^Department of Biochemistry, Faculty of Pharmaceutical Sciences, Fukuoka University, Fukuoka, Japan; ^2^Department of Biochemistry, School of Medicine, Fujita Health University, Toyoake, Japan

**Keywords:** aromatase, steroid hormone, sexual differentiation, estrogen, chicken ovalbumin upstream promoter transcription factor, nuclear receptor

## Abstract

An important function of aromatase in the brain is conversion of testosterone secreted from the testis into estradiol. Estradiol produced in the brain is thought to be deeply involved in the formation of sexually dimorphic nuclei and sexual behavior as a neurosteroid. We analyzed the brain-specific promoter to elucidate the control mechanisms of brain aromatase expression that may be highly involved in sexual differentiation of the brain. The 202-bp upstream region of the brain-specific exon 1 has three types of *cis*-acting elements, aro-AI, AII, and B. We isolated ARP-1 as an aro-AII-binding protein by yeast one-hybrid screening from a cDNA library of mouse fetal brains. ARP-1 is a member of the nuclear receptor superfamily and functions as an orphan-type transcription factor. ARP-1 protein synthesized *in vitro* showed the same binding property to the aro-AII site as nuclear extract from fetal brains. To determine how the promoter is involved in brain-specific transcription of the aromatase gene, we first detected the *in vivo* occupancy of the aro-AII site by ARP-1 using chromatin immunoprecipitation assays. Diencephalic regions of fetal brains at embryonic day 16 were analyzed, which revealed ARP-1 recruitment to the aro-AII site. To analyze the effects of ARP-1 on transcriptional regulation of the brain-specific aromatase promoter, a luciferase reporter plasmid driven by the brain-specific promoter was transfected into CV-1 cells together with a plasmid expressing ARP-1 protein. These analyses revealed that ARP-1 induced promoter activity in a dose-dependent manner. Furthermore, to determine whether ARP-1 is required for aromatase expression in neurons, ARP-1 knockdown was conducted in neuronal cell primary culture. Knockdown of ARP-1 significantly suppressed the increase in aromatase mRNA observed in cultured neurons. These results indicate that ARP-1 is involved in the transcriptional regulation of the brain-specific promoter of the aromatase gene.

## Introduction

The physiological functions of neurosteroids have been investigated in many laboratories (1–3). Neurosteroids facilitate a wide variety of biological activities in the brain either through the action of a canonical nuclear receptor or through interaction with membrane-bound receptors (4–6). The neurosteroid estrogen has been proposed to play critical roles in a variety of reproductive behaviors. Aromatase, also called estrogen synthase, is mainly expressed in the gonads (7–9) and brain (10–13) in rodents. We have shown that aromatase plays an important role in the formation of morphological, neuroendocrinological, and behavioral sex differences. In fact, an experimental animal model of estrogen deficiency was generated in mice by targeted disruption of the aromatase gene (14–16), and the roles of estrogen in reproductive behaviors were extensively investigated (17–19). In our previous study, transgenic mice specifically expressing human aromatase in the brain were generated and crossed with aromatase knock-out (ArKO) mice, resulting in the creation of mice with brain-specific recovery of estrogen production (ArKO/bsArTG) (20). The ArKO/bsArTG mice exhibited significant restoration of impaired behaviors, suggesting that brain-restricted expression of aromatase is sufficient for the display of reproductive behavior. Thus, expression of aromatase in the brain is suggested to be essential for reproductive behavior in mice.

Transcription of the aromatase gene is governed by multiple tissue-specific promoter regions. In the brain, expression of the aromatase gene varies depending on the developmental stage, with a transient peak during the perinatal period, which is consistent with the critical period known as neonatal imprinting of sexual differentiation. We have identified a brain-specific exon 1 in human and mouse aromatase genes, and its use was restricted to neurons by alternative splicing of the multiple exons 1 (21–26). The promoter analyses revealed that the 202-bp upstream region of the mouse brain–specific exon 1 has strong promoter activity in primary culture of diencephalic neurons from fetal mouse brains. We have shown the functions of *cis*-acting elements responsible for the brain-specific spatiotemporal expression of the mouse aromatase gene. The 202-bp upstream region has three *cis*-elements: aro-AI (Arom-Aα), aro-AII (Arom-Aβ), and aro-B (Arom-B) (27, 28). Our previous study indicated the homeodomain-containing transcription factor Lhx2 as a binding protein to the aro-B site and a potential transcriptional regulator of brain-specific expression of the aromatase gene (28). Lhx2 can mediate transcriptional activity of the brain-specific aromatase gene and exhibits a transient peak during the perinatal period. In concordance with previous findings, it is highly likely that unidentified transcription factors, which bind to other *cis*-acting elements, including aro-AI and aro-AII, are also involved in the transcriptional regulation of aromatase in the brain.

In the present study, we provide evidence that a member of the nuclear receptor super family, ARP-1, can bind to the aro-AII site of the brain-specific promoter 1f and positively regulate aromatase expression.

## Materials and Methods

### Yeast One-Hybrid Screening

The Matchmaker One-Hybrid System was used to isolate the cDNA encoding a protein that binds to the aro-AII element of the brain-specific promoter of the mouse aromatase gene. The procedures were performed according to the manufacturer's protocols (Clontech, Mountain View, CA, USA). Four tandem repeats of double stranded aro-AII (5′-TTATGTTGGCCCCTGACATATATATT-3′) nucleotides were subcloned into the upstream regions of the minimal promoters of pHISi-1 and pLacZ reporter plasmids. These plasmids were then linearized and transformed for integration into a YM4271 yeast genome to generate reporter yeast strains that were designated as YM4271/aro-AII-His and YM4271/aro-AII-LacZ. YM4271/aro-AII-His was further checked for growth on medium lacking histidine (His^−^ medium) in the presence of 45 mM 3-amino-1,2,4-triazol (3-AT). The YM4271/aro-AII-His yeast strain was transformed with a MATCHMAKER cDNA library constructed from embryonic day 17 (E17) mice and subsequently cloned into the vector pACT2 (Clontech). The transformed yeast colonies (~1.5 × 10^6^) were screened, and three positive transformants, which were grown on SD medium plates lacking histidine and leucine with 45 mM 3-AT, were isolated. To exclude pseudo-positive clones, plasmids were recovered from selected clones and rescreened by transforming them into YM4271/aro-AII-LacZ cultures on SD medium plates lacking uracil and leucine. The filter replica method—using 5-bromo-4-chloro-3-indolyl-β-D-galactopyranoside (40 μg/ml)—was used to measure β-galactosidase activity according to the manufacturer's protocols. DNA sequences of plasmids from three individual positive blue colonies were sequenced and subsequently analyzed for homology using a BLAST DNA search.

### Gel Shift Assay

Gel shift assays were carried out as described previously (29). For the gel shift assay, the 5′-protruding ends of the double-stranded aro-AII probe (5′-gTTATGTTGGCCCCTGACATATATATT-3′/5′-gAATATATATGTCAGGGGCCAACATAA-3′) were labeled with [α-^32^P] dCTP using a Klenow fragment of DNA polymerase. Five micrograms of nuclear protein or aliquots of proteins synthesized *in vitro* were mixed with the DNA probes and incubated for 20 min on ice. The reaction mixtures were analyzed using a 5% polyacrylamide gel. The synthetic mutant oligonucleotides, AIIM1 and AIIM3, were also described in a previous paper (29). For competition experiments, a 200-fold molar excess of unlabeled nucleotides was added to the reaction mixture. For supershift experiments, an anti-ARP-1 antibody (Santa Cruz Biotechnology, Santa Cruz, CA, USA) or anti-COUP-TFI (Chicken Ovalbumin Upstream Promoter-Transcription Factor I) antibody (Santa Cruz Biotechnology) was used to specifically recognize corresponding isoforms.

Plasmid constructs bearing ARP-1 that were suitable for *in vitro* translation were prepared as follows. ARP-1 cDNA obtained was subcloned into a pCI-neo vector (Promega, Madison, WI, USA), resulting in pCI-neoARP-1. The plasmid was linearized at the 3′ end of the coding region and transcribed by T7 RNA polymerase (Takara, Kyoto, Japan). The resulting ARP-1 mRNA was verified by 1% agarose gel electrophoresis. ARP-1 mRNA was translated using an *in vitro* protein synthesis kit (Promega) with rabbit reticulocyte lysate.

### Animals

All experimental procedures using animals were approved by the Committee for Animal Experiments of Fukuoka University (reference no. 1705049).

### Chromatin Immunoprecipitation (ChIP) Assay

**Figure 1:**
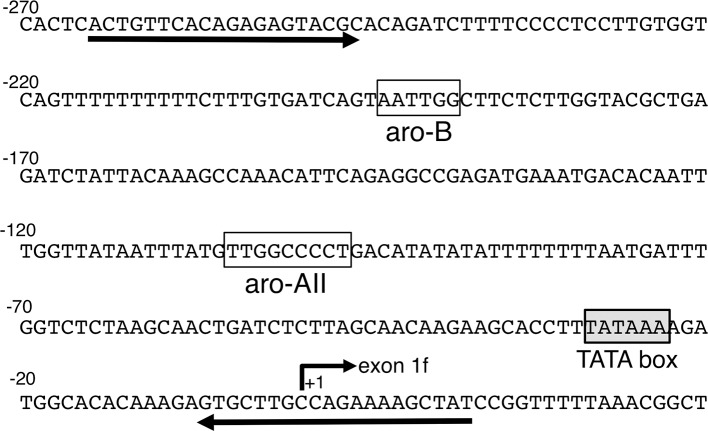
Brain-specific exon 1 and its promoter region in the mouse aromatase gene. The number +1 corresponds to a potential transcription start site. A TATA box is shown in the shadowed box. The open boxes indicate the aro-AII and aro-B sites found in previous studies (27). The two primers used in the chromatin immunoprecipitation assay are also indicated in the figure by the arrows.

The chromatin immunoprecipitation assay was carried out using a ChIP assay kit (Upstate Cell Signaling Solutions, Charlottesville, VA, USA) as described previously. The diencephalic regions of E16 mouse fetal brains were treated with 1% formaldehyde to cross-link proteins to DNA. Then, the samples were homogenized in lysis buffer and sonicated to yield an average DNA size of 500 bp. Sonicated extracts were precleared with protein G-agarose/salmon sperm DNA (Upstate Cell Signaling Solutions) and divided into two fractions. Then, 5 μg of non-immunized goat immunoglobulin G (preimmune IgG) or anti-ARP-1 antibody (Santa Cruz Biotechnology) was applied. The immunoprecipitated products were eluted, and DNA–protein complexes were dissociated by heating at 65°C. The resulting DNA fraction was purified by phenol/chloroform extraction and ethanol precipitation and subsequently subjected to PCR amplification using the following aromatase gene-specific primers: MB-AR-N1, 5′-TCACTGTTCACAGAGAGTAC-3′; MB-AR-0R, 5′-ATAGCTTTTCTGGCAAGCAC-3′ (Figure 1).

### Aromatase Gene Promoter Assay Using a Luciferase Reporter

CV-1 and HepG-2 cells were cultured in Dulbecco's modified Eagle's medium (DMEM) supplemented with 10% fetal bovine serum, 100 units/ml of penicillin, and 100 μg/ml of streptomycin. Luciferase reporter plasmids were constructed by cloning the fragments of brain-specific promoters into the pGL3-Basic luciferase vector (Promega, Madison, WI). To obtain fragments of the promoter region, we amplified the fragments by polymerase chain reaction (PCR) using mouse genomic DNA as a template and oligonucleotide pairs; the brain-specific promoter region of the mouse aromatase gene was amplified with the following primer pair: MB-AR-N1 (5′-TCACTGTTCACAGAGAGTAC-3′) and MB-AR-1R (5′-GGACTCTTGAAGATGGTGAG-3′), and the mouse apolipoprotein AI promoter region was amplified with the following primer pair: mo-apoA1-2 (5′-TGGGACCCCTGGAGTCTGC-3′) and mo-apoA1-R1 (5′-GGACGCTCTCCGACAGTCT-3′). The PCR products were subcloned into the SmaI site of the pGL3-Basic plasmid (Promega), resulting in the pGL3aroBr and pGL3apoAI plasmids. The cDNA clone of mouse ARP-1 was subcloned into the Bam HI and Not I sites of the p3XFLAG-myc-CMV-26 expression vector (Sigma-Aldrich, St. Louis, MO, USA), resulting in the pFLAG-ARP-1 expression plasmid.

The pFLAG-ARP-1 expression plasmid (50, 100, 250, or 500 ng), 500 ng of reporter plasmid, and 50 ng of phRluc-TK control vector (Promega) were mixed with 50 μl of antibiotic-free DMEM containing 8 μl of Plus Reagent (Invitrogen) and incubated for 15 min at room temperature. Next, 50 μl of antibiotic-free DMEM containing 2 μl of Lipofectamine Reagent (Invitrogen) was added to the mixture and incubated for 15 min at room temperature. The mixture was added onto CV-1 cell monolayers preincubated under serum-free conditions. After 5 h of incubation, the DNA–liposome complex was replaced with the complete medium and cultured for 48 h. For the reporter assay with HepG2 cells, 100, 250, or 500 ng of pFLAG-ARP-1 expression plasmid, 500 ng of pGL3aroBr or pGL3apoAI plasmid, and 50 ng of phRluc-TK control vector (Promega) were mixed and transfected in cells according to the procedure described above. The cells were solubilized with 150 μl of Passive Lysis Buffer (Promega). Promoter activity was measured using a Dual-Luciferase Reporter Assay system (Promega) according to the manufacturer's protocol. Each experiment was performed in duplicate and repeated at least three times.

### ARP-1 Knockdown of Fetal Neuronal Cells

Diencephalic neurons were prepared from E13 mouse fetal brains according to Abe-Dohmae et al. (30), and the cells were used to analyze the effects of ARP-1 knockdown on diencephalic aromatase expression. The neuronal cells were cultured in DF medium (50% DMEM and 50% Ham's F12 medium, supplemented with 5 μg/ml of insulin, 5 μg/ml of human transferrin, 5 ng/ml of sodium selenite, 20 nM of progesterone, 100 units/ml of penicillin G, and 100 μg/ml of streptomycin sulfate) in a poly-L-Lys-coated 12-well-plate (1.5 × 10^6^ cells/well). Twenty-four hours after starting the culture, transfection was performed using the HiPerFect Transfection reagent (Qiagen, Valencia, CA). For transfection, the cells were washed twice with antibiotic-free DF medium and subsequently incubated in 1.2 ml of antibiotic-free DF medium containing 25 nM Silencer Select siRNA (siRNA ID: s102050) according to the manufacturer's instructions. Forty-eight hours after starting the transfection, the cells were harvested to prepare separate RNA and protein samples. Total RNA was prepared using the TRIzol regent (Invitrogen) and was then analyzed by RT-qPCR. The reduction in ARP-1 protein was estimated by western blot analysis using an anti-ARP-1 antibody (Santa Cruz Biotechnology) and an anti-β-actin antibody (Proteintech, Rosemont, IL, USA). For the western blotting analysis, the cells were washed with PBS, lysed in RIPA buffer [150 mM NaCl, 1% Nonidet P-40, 0.5% sodium deoxycholate, 0.1% sodium dodecyl sulfate, and 50 mM Tris (pH 7.6)], then centrifuged at 14,000 × g for 10 min. The protein concentration was determined using a BCA protein assay kit (Bio-Rad, Hercules, CA, USA). Five micrograms of the protein lysate was separated on a 9% sodium dodecyl sulfate-polyacrylamide gel, then electro-transferred to a PVDF membrane. The membrane was blocked in Blocking One solution (Nacalai Tesque, Kyoto, Japan) for a duration ranging from 1 h to overnight, then incubated with anti-ARP-1 antibody at a dilution of 1:5,000 or anti-β-actin antibody at a dilution of 1:5,000 for 1 h at room temperature. The membrane was then incubated with the appropriate peroxidase-conjugated secondary antibody at a dilution of 1:20,000 (Vector Laboratories, Burlingame, CA, USA) for 1 h at room temperature. Can Get Signal Immunoreaction Enhancer Solution (TOYOBO, Osaka, Japan) was used to dilute the primary and secondary antibodies. Chemiluminescence was detected using Immobilon Western Chemiluminescent HRP Substrate (Millipore, Billerica, MA, USA). The immunoreactive signals were visualized and quantified using a ChemiDoc XRS instrument (Bio-Rad).

### RT-qPCR

The reverse transcription reaction was performed using an AffinityScript QPCR cDNA Synthesis Kit (Agilent Technologies, Santa Clara, CA, USA). Briefly, total RNA (3 μg) was reverse transcribed using random primers according to the manufacturer's instructions. cDNA aliquots were used for quantitative PCR analysis. Real-time PCR was performed using TaqMan probes with Brilliant II QPCR Master Mix (Agilent) according to the manufacturer's instructions. TaqMan Gene Expression Assay reagents (Thermo Fisher Scientific, Waltham, MA, USA) for aromatase (Assay ID: Mm00484049_m1) and β-actin (Assay ID: Mm01205647_g1) were used as TaqMan probes. Real-time PCR was performed using a two-step cycling protocol consisting of 45 cycles of 20 s at 95°C and 60 s at 60°C on an Mx3000P QPCR System (Agilent). All reactions included controls lacking the template. After the reactions, the Ct values were determined using fixed-threshold settings. The ΔΔCT method was used to determine the mRNA fold change, which was normalized to β-actin mRNA level. Each experiment was performed in duplicate and repeated at least three times.

### Statistical Analysis

The results obtained from triplicate experiments are expressed as the mean ± S.E.M. All data analyses were performed using JMP® software (SAS institute Inc., Cary, NC, USA). Statistical analysis of the data was performed using one way ANOVA with *post-hoc* Tukey–Kramer correction, and differences were considered significant if the *p* < 0.05.

## Results

### Identification of a Protein That Interacts With the aro-AII Site of the Brain-Specific Promoter 1f of the Aromatase Gene

To obtain a potential transcription factor that binds to the aro-AII sequence, we performed screening of an E17 mouse cDNA library using reporter yeast strains, designated as YM4271/aro-AII-His and YM4271/aro-AII-Lac.

Three positive clones were obtained from a one-hybrid screening of the cDNA library from E17 mice. Comparison of the sequences with GeneBank data using a BLAST DNA search revealed a sequence that was 100% identical to the previously identified transcription factor apolipoprotein AI Regulatory Protein 1 (ARP-1, also called COUP-TFII). These clones did not cover the full length of the ARP-1 protein, and an even longer clone was lacking nine amino acids of the N-terminus.

### ARP-1 Protein Binds to the aro-AII Site

**Figure 2:**
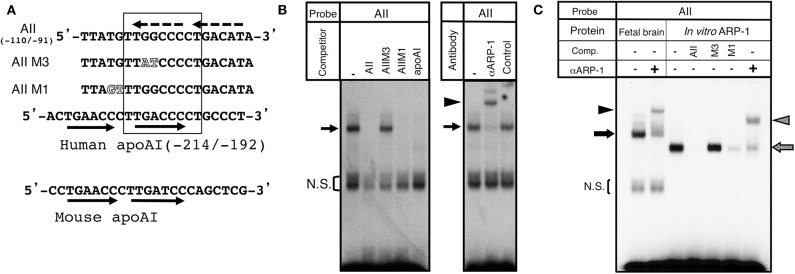
Identification of ARP-1 as a candidate binding protein of the aro-AII site. **(A)** The aro-AII-binding protein recognized the sequence “TTGGCCCCT,” which is shown inside the square. The wild-type and mutant oligonucleotide probes, AII, AIIM1, and AIIM3, are shown. Mutations introduced into the aro-AII oligonucleotide are shown by outlined characters. The nucleotide sequence of the ARP-1 binding site in the promoter region of the human and mouse ApoAI gene is also shown in the figure. Tandem repeats of human and mouse ApoAI oligonucleotides are shown in the oligonucleotide by arrows. The box indicates the portion of nucleotides essential for ARP-1 binding as described in our previous report. The putative tandem repeats in the AII probe are shown by broken arrows. **(B)** A gel shift assay was performed using 5 μg of nuclear protein. A 200-fold molar excess of unlabeled probe was used in the competition assay. The specific signals are indicated by the arrow on left side. The bracket shows the non-specific signals (N.S.). **(C)** For supershift assays, nuclear protein was incubated with 2 μg of the indicated antibody on ice for 30 min before the addition of a radiolabeled probe. The probe/nuclear protein complex and supershift signals are indicated by the arrow and arrowhead, respectively, on the left side.

To assess ARP-1 binding activity to the aro-AII sequence, we carried out gel shift analyses with nuclear extracts prepared from the diencephalic region of E15 mouse brains. The sequence required for aro-AII to interact with the binding protein is shown in Figures 1,2A. In a gel shift assay using an AII probe that included the TTGGCCCCT sequence of the promoter region, the nuclear protein formed a specific mobility-shifted complex on the AII probe, and this complex did not form under competition with a 200-molar excess of an unlabeled AII probe. The aro-AII-binding protein recognized a nine-base-pair sequence “TTGGCCCCT” as shown in Figure 2A. When a mutation was introduced into this nine-base-pair sequence, the interaction with the protein was lost. As previously shown by our group (29) and indicated in Figure 2A, an oligonucleotide with a mutant introduced inside the “TT*AT*CCCCT” (AIIM3) sequence did not compete with the complex, but an oligonucleotide mutated outside the sequence (AIIM1) could compete with the complex (Figure 2B, left panel). ARP-1 was found to be a transcriptional regulator of apolipoprotein A1 (31). A gel shift assay was performed using the ARP-1-binding site present in the promoter sequence of mouse apolipoprotein as a competitor (Figure 2B, left panel). A similar competition was also observed when the sequence present in the apolipoprotein gene was used as a competitor oligonucleotide. Moreover, the ARP-1 antibody super-shifted the complex formed by aro-AII and the nuclear factor on the gel shift assay (Figure 2B, right panel). To further conform the binding ability of ARP-1 to aro-AII, similar gel shift experiments were conducted using ARP-1 protein synthesized *in vitro* from the cDNA obtained by one-hybrid screening. As shown in Figure 2C, the ARP-1 synthesized *in vitro* had a slightly higher mobility complex than the nuclear protein, presumably because of the nine missing N-terminal amino acids from the wild-type ARP-1. The binding properties of the synthesized ARP-1 protein were apparently identical to those of the nuclear protein in brain extract. These results suggested that the binding protein of aro-AII is ARP-1.

### ARP-1 Binds to aro-AII in the Brain-Specific Promoter 1f Region of the Mouse Aromatase Gene *in vivo*

**Figure 3:**
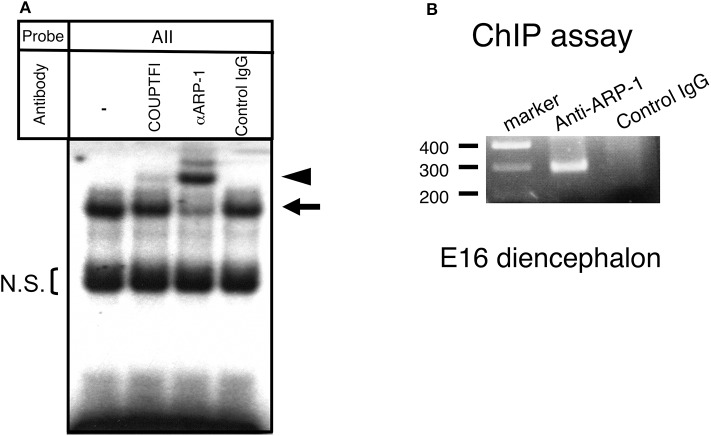
ARP-1 protein binds to the aro-AII sequence *in vivo*. **(A)** A gel shift assay was performed using 5 μg of nuclear protein or an aliquot of *in vitro* synthesized ARP-1 protein. For supershift assays, nuclear protein was incubated with 2 μg of the indicated antibody on ice for 30 min before the addition of a radiolabeled probe. The probe/nuclear protein complex and supershift signals are indicated by the arrow and arrowhead, respectively, on the left side. The bracket shows the non-specific signals (N.S.). A 200-fold molar excess of unlabeled probe was used in the competition assay. The probe/ARP-1 complex and supershift signals are indicated by the hatched arrow and arrowhead, respectively, on the right side. **(B)** A chromatin immunoprecipitation assay confirmed that ARP-1 could associate with the aro-AII site in the fetal mouse brain *in vivo*. Fresh diencephalic regions of E16 mouse brains were treated with 1% formaldehyde. The fixed tissues were dissolved, and the DNA was sheared and immunoprecipitated with anti-ARP-1 antibody or preimmune IgG. The recovered genomic DNA was subjected to PCR with primers specific for the mouse aromatase gene as shown in Figure 1.

In the E15 mouse brain, COUP-TFI, a protein with high homology and similar binding sites to ARP-1, is also observed. Thus, considering the possibility that COUP-TFI can also occupy the aro-AII site, we conducted a supershift analysis with specific antibodies by gel shift assay using fetal brain extract. As shown in Figure 3A, when the anti-ARP-1 antibody was added, a clear supershift band was observed, whereas only a faint supershift band was visible with the anti-COUP-TFI antibody. These results suggested that the majority of the protein that binds at the aro-AII site in the E15 fetal brain is ARP-1. To confirm that ARP-1 binds to the aro-AII site in the promoter 1f of the aromatase gene *in vivo*, we conducted a ChIP-PCR assay using the diencephalic region of E16 fetal mouse brains. As shown in Figure 3B, anti-ARP-1 IgG immunoprecipitated the promoter region containing the aro-AII site, resulting in an amplified DNA product on ChIP-PCR. In contrast, no PCR product was observed in the assay using control IgG. The results suggested that ARP-1 binds to the aro-AII *cis*-element in the 1f promoter region *in vivo*.

### Regulatory Function of ARP-1 for the Brain-Specific Promoter 1f of the Aromatase Gene

**Figure 4:**
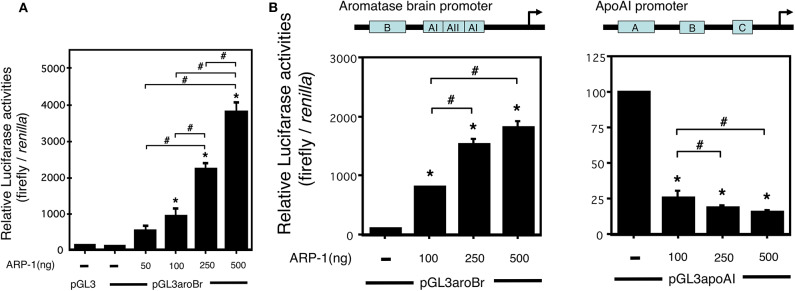
ARP-1 increases brain-specific promoter activity of the mouse aromatase gene. **(A)** ARP-1 increases the promoter activity in a dose-dependent manner. The reporter plasmid (500 ng), pGL3aroBr, was co-transfected with the indicated amounts of pFLAG-ARP-1 plasmid (50, 100, 250, and 500 ng) and 50 ng of the internal control plasmid into CV-1 cells. The total amount of expression plasmid was adjusted to 500 ng with an empty plasmid (p3XFLAG-myc-CMV-26). The cells were harvested after 48 h, and a Dual-Luciferase Reporter Assay was carried out as described in the Materials and Methods section. **(B)** Effects of ARP-1 on the activity of aromatase and apolipoprotein AI promoters in HepG2 cells. A Dual-Luciferase Reporter Assay was conducted as described in **(A)**. The mean ± SEM of at least three independent experiments is shown in the figure. One-way ANOVA showed a significantly different distribution (*p* < 0.0001 for **(A,B)**. The *p*-value of the Tukey–Kramer test is indicated with the symbols as follows. Asterisks indicate statistically significant differences in relative promoter activity between the empty plasmid alone and that after co-transfection with the ARP-1 expression plasmid (*p* < 0.05). Pound signs indicate statistically significant differences in comparisons between indicated pairs (*p* < 0.05).

To determine the regulatory effects of ARP-1 on the transcription from the promoter 1f of the aromatase gene, we performed a luciferase reporter assay. A luciferase reporter plasmid, pGL3aroBr, was transfected into CV-1 cells together with increasing amounts of ARP-1 expression plasmid. As shown in Figure 4A, ARP-1 dose-dependently enhanced luciferase reporter activity, reflecting the transcriptional activity of the promoter 1f, showing an ~40-fold enhancement with 500 ng of ARP-1 expression plasmid. ARP-1 has been reported to repress the expression of the apoAI gene in human hepatoma-derived HepG2 cells (31). To examine whether the effects of ARP-1 on activity of the brain-specific aromatase promoter depend on the properties of cell lines, we conducted a luciferase reporter assay using HepG2 cells. ARP-1 repressed the promoter activity of the pGL3apoAI-containing apoAI promoter and enhancer in HepG2 cells. ARP-1, however, still showed the ability to enhance promoter activity of the brain-specific aromatase in HepG2 cells (Figure 4B).

**Figure 5:**
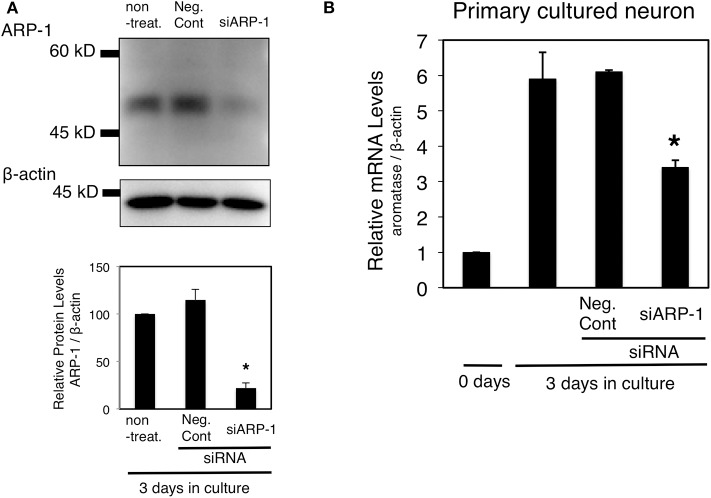
Effect of ARP-1-targeting siRNA on expression of the endogenous aromatase gene in primary cultured neural cells. **(A)** Diencephalic neurons were transfected with ARP-1-targeting siRNA and cultured for 48 h, and then the lysates were subjected to western blotting with an anti-ARP-1 antibody and an anti-β-actin antibody (top panel). The amount of ARP-1 protein is quantitated and expressed as the relative ARP-1/β-actin value (lower panel). The mean ± SEM of three independent experiments is shown in the figure. One-way ANOVA showed a significantly different distribution (*p* < 0.0002). The *p*-value of the Tukey–Kramer test is indicated with the symbol as follows. Asterisks indicate statistically significant differences in ARP-1 protein levels between the ARP-1 siRNA and negative control siRNA groups (*p* < 0.05). **(B)** Effect of ARP-1 knockdown on the aromatase mRNA level in neural cells. Diencephalic neurons were prepared as in **(A)**, and total RNA was extracted from the cultured cells. The total RNA was analyzed by RT-qPCR to determine the amount of aromatase mRNA as described in the Materials and Methods section. The results are presented as the mean ± SEM of three independent experiments. One-way ANOVA showed a significantly different distribution (*p* < 0.0002). The *p*-value of the Tukey–Kramer test is indicated with the symbol as follows. Asterisks indicate statistically significant differences in aromatase mRNA levels between the ARP-1 siRNA and the negative control siRNA groups (*p* < 0.05).

Previous studies have shown that *in vitro* cultured fetal diencephalic neurons can express aromatase mRNA (30). The mRNA expression level increased in a time-dependent manner for 3 days in E13 neurons prepared from the fetal diencephalon (32). To determine whether ARP-1 is required for aromatase expression in neurons, ARP-1 knockdown followed by real-time PCR was conducted. Addition of an siRNA against ARP-1 led to a significant decrease in the ARP-1 protein level in primary cultured neurons (Figure 5A). The aromatase mRNA level on day 3 in cultured neural cells was increased by ~6-fold compared with the level on day 0 (in primary cultures derived from E13 mice). As shown in Figure 5B, ARP-1 knockdown by ARP-1-targeting siRNA decreased the increment of the aromatase mRNA level in cultured nerve cells by 43%.

## Discussion

We have previously analyzed the transcriptional mechanisms that regulate the brain-specific expression of the mouse aromatase gene, revealing three *cis*-elements in the promoter region. In the present study, we demonstrated that ARP-1, a member of the nuclear receptor superfamily, binds to the aro-AII site and is a positive transcriptional modulator of brain-specific expression of the aromatase gene. Cloning of ARP-1, also called COUP-TFII, revealed that it is highly homologous to COUP-TFI (33), and it was found to repress transcription of the apolipoprotein A1 gene via site A in its promoter region (31). The gel shift assays indicated that the ARP-1 protein was involved in aro-AII site binding. ARP-1 has been shown to bind to a wide spectrum of response elements encompassing AGGTCA direct repeats with various spacings, while it has the highest affinity for direct repeats of AGGTCA with one nucleotide spacing (DR1 element) (34). Our previous study revealed the essential nucleotide sequence for binding to the aro-AII site (29). The putative DR-1 was found in the nucleotide positions −110/−91 of the promoter region, although no apparent sequence of the direct repeat was observed within the essential nucleotide sequence for binding (Figure 2A). However, aro-AII with a mutation in one of the repeats (5′-ATGTCA to 5′-ATGcaA) retained binding activity with nuclear protein (29). Additional analysis is necessary to determine of the features of ARP-1 binding to the aro-AII site.

Expression of the aromatase gene in the mouse brain exhibits a transient peak during the perinatal period (35). A similar transient peak of expression was also observed in the *in vitro* system, even if diencephalic tissues were dissociated and dispersed as individual neural cells (32). In this study, the luciferase reporter gene assay showed that ARP-1 activated transcription of the reporter gene in a dose-dependent manner (Figure 4). A decrease in ARP-1 protein caused by RNA interference reduced the spontaneous increase in aromatase mRNA levels in cultured neural cells from the diencephalic region of the fetal mouse brain (Figure 5). Moreover, ChIP-PCR analysis indicated that endogenous ARP-1 protein bound to the aro-AII site *in vivo* (Figure 3). These results suggest that ARP-1 is a transcription factor that positively regulates aromatase expression in the mouse brain via specific binding to the aro-AII site on the promoter 1f of the gene. ARP-1 can either positively or negatively modulate the expression of downstream genes through different mechanisms (34, 36, 37). ARP-1 exhibits positive regulation of the brain-specific promoter of the aromatase gene in both in CV-1 and HepG2 cells, suggesting that the function of ARP-1 in transcriptional regulation may depend on the target promoter context. Earlier, we demonstrated that a *LacZ* reporter gene driven by the −6.5-kb promoter region of the exon 1f showed almost the same spatiotemporal expression as the endogenous aromatase gene using transgenic mice (38). The −0.2-kb promoter region, however, partially reproduced endogenous aromatase expression, while the reporter gene was also observed in the extra-brain tissues (our unpublished observations (39). Moreover, a mutation introduced into the aro-AII site of the −6.5-kb promoter in the *LacZ* reporter caused a significant decrease in brain expression, and ectopic expression was observed. These results suggest that the aro-AII element is necessary, but not sufficient, for spatiotemporal expression of the aromatase gene in the brain.

ARP-1 is predominantly expressed in mesenchymal cells during organogenesis. The spatiotemporal expression of mouse COUP-TFs, including ARP-1, in the brain has also been determined (40). ARP-1 is first observed at approximately E8.5, peaks at E14–E15, and then decreases after birth (41, 42). In our previous report, an analysis of aro-AII binding activity in the brain at the perinatal, neonatal, and adult stages showed that the binding activity to nuclear protein was extremely reduced at the adult stage (29). These results are very consistent with the endogenous ARP-1 expression pattern. ARP-1 in the brain is predominantly localized in the diencephalon (43, 44, 61), while high expression of ARP-1 is also detected in the amygdaloid nucleus (45–47). Aromatase expression is initially observed at E13–14 in the mouse diencephalon; it subsequently increases at E16–17 and then decreases gradually to the adult expression level, with a transient peak during the perinatal period. The expression patterns of aromatase and ARP-1 in the brain are not completely identical, but these patterns are indeed overlapping.

Homozygous ARP-1 mutant mice exhibit various morphological abnormalities, such as defective angiogenesis and vascular remodeling, which result in death by E9.5 because of severe hemorrhage and edema in the brain and heart (48). Moreover, no homozygous ARP-1 mutant mice are detected at E11.5, and even if born, two-thirds of heterozygous mutants die before weaning (48). Recently, ventral forebrain–specific disruption of the ARP-1 gene has been shown to cause agenesis of the basomedial amygdala nucleus, indicating that the ARP-1 gene directs neuronal progenitor cells to generate the basomedial amygdala nucleus (47). Moreover, mice with a ventromedial hypothalamus–specific ARP-1 mutant gene have also been generated using *Cre* driven by the Ad4BP/SF-1 promoter (49). Unfortunately, the homozygous hypothalamus–specific mutants displayed embryonic lethality. New model animals may be needed to determine the interaction of ARP-1 with the brain-specific aromatase gene *in vivo*.

Estradiol-17β has been reported to serve as a regulatory factor that controls the expression of aromatase and its enzymatic activity in the brain (50–55). Yilmaz et al. investigated the brain-specific promoter using a murine hypothalamic neuronal cell line. They showed that estradiol regulated brain-specific aromatase transcription, and the −200/−1 region of promoter 1f participated in the estrogen responsiveness. Typical palindromic estrogen–responsive elements or their half-sites were not identified in the promoter region, while two AP-1-binding sites in the region might be essential for induction of transcriptional activity by estradiol. Binding of estrogen receptor α and c-Jun to AP-1-binding sites might positively regulate the transcriptional activity of the promoter 1f (56). Bulun and colleagues also reported functions of progesterone and glucocorticoid receptors as negative and positive regulators, respectively, for activity of the promoter 1f (57, 58). The inverse regulation of 1f promoter activity by progesterone and glucocorticoid is interesting considering that progesterone and glucocorticoid response elements share the same sequence. Interestingly, Cisternas et al. reported that estradiol increased aromatase mRNA and protein levels only in female neuronal cells from the anterior amygdala of embryonic brains (59). Estrogen receptor β is involved in the incremental expression of aromatase by estradiol and binds 5α-androstane-3β,17β-diol as a ligand in addition to estradiol (60). The aromatase expression patterns, which are specific for developmental stages or brain regions, may be produced using functional complexes consisting of multiple transcription factors and various hormonal factors. The local production of estrogen in the brain may serve as a neurosteroid during organization of the neuronal network, including sexually dimorphic nuclei, and in motivation/activation of sexual behaviors.

Taken together, these data support that ARP-1 is a transcription factor that regulates aromatase expression in the brain by binding to the aro-AII site on the promoter. Further studies on the transcription factors and their cofactors should be performed to elucidate the molecular processes of the spatiotemporal expression of brain aromatase and the biological processes of the organization and activation effects of estrogen.

## Data Availability Statement

The datasets generated for this study are included in this published article.

## Author Contributions

SH and NH designed this work and wrote the manuscript. SH performed the experiments and analyzed the data.

## Conflict of Interest

The authors declare that the research was conducted in the absence of any commercial or financial relationships that could be construed as a potential conflict of interest.
